# The Treatment of Prolapse of the Uterus, Cystocele and Elongation of the Cervix

**Published:** 1909-05-29

**Authors:** 


### GYNÆCOLOGY.

### THE TREATMENT OF PROLAPSE OF THE UTERUS, CYSTOCELE AND ELONGATION OF THE CERVIX.

Much misconception exists as to the modern operative treatment of uterine prolapse and its attendant disorders. Hysterectomy by itself will not cure the vaginal eversion and cystocele which accompany prolapse, for these conditions are apt to recur after the uterus has been removed, and the symptoms in this case will be much the same as if the uterus were present. In those cases which hysterectomy has apparently cured, an explanation will be found in some septic process set up in the bases of the broad ligaments, by which the vaginal walls are fixed in the pelvis—in other words, by a pelvic cellulitis. This process cannot be regarded as an ideal method of cure; for it is accidental, it cannot be controlled, and it may prove fatal. Ventro-fixation too by itself is no cure for cystocele or elongated cervix, for these will occur although the fundus is firmly fixed to the abdominal wall. In very mild cases, no doubt, ventro-fixation produces marvellous results; but mild cases are just those which can be cured by any of the simple plastic operations on the pelvic floor which are practically devoid of risk. The abdominal route is quite unjustifiable for mild cases of prolapse, say, of the first degree, even if ventro-fixation were an ideal operation.

On the other hand ventro-fixation and even hysterectomy, combined with properly planned plastic operations on the pelvic floor, may prove eminently useful; but their use for the cure of prolapse and cystocele must be strictly limited in this way. The principle upon which a severe case of prolapse with cystocele and cervical elongation must be attacked is that of restoring as far as possible the uterus, vaginal walls, bladder, rectum, and pelvic floor to their normal relations to one another, and not by any artificial method of slinging up the uterus. To carry out this principle several distinct operative procedures are necessary, all of which are mutually dependent and essential. These procedures naturally depend upon the degree of uterine prolapse, of cervical elongation, of cystocele, and of rectocele. In general it may be said that if the uterus is congested and there is endometritis, the cavity must be first dilated and curetted with a sharp instrument. If the cervix is elongated it must be amputated. If there is a cystocele the bladder must be dissected free from the vaginal wall and pushed up into its normal position, and sutures must be placed to keep it there. Redundant vaginal wall is then cut away and the wall sutured. If there is a rectocele the rectum must be similarly treated. Finally, the perineum must be restored and extended anteriorly. These five operations are absolutely necessary in most cases of any severity, and may be all carried out at one sitting by a moderately rapid operator. There are a few details of these operations which require to be amplified; but they are not in any way novel; in fact, they have all been performed, lauded, and condemned for the last quarter of a century or longer. We say "condemned" because there are operators who believe them useless for severe cases.

These operations must all be performed on each case as a rule; they must be carried out step by step, with the strictest attention to asepsis, technique, preparation, and after-treatment. If they are regarded as minor surgery, and any essential is omitted, the result will probably be absolute failure. A mere perineorrhaphy is quite useless to cure prolapse with cervical elongation and cystocele, and, further, an amputation of the cervix will not cure a prolapse if no plastic work is done.

To secure asepsis, cleansing of the cervix, vagina, and external genitals must be carried out in the same way as for a laparotomy—namely by shaving, ether soap and hot water, followed by an efficient antiseptic. The vagina cannot be made aseptic by a douche or douches; it must be scrubbed with absorbent wool, soap, and antiseptic. Boiled gloves should be worn by the operator, for the bare hands will easily infect sutures and ligatures. No douches should be given afterwards, for they are almost certain to introduce sepsis from without, and even if they could be given aseptically they are useless and unnecessary. Suture materials must be most carefully prepared, and for all sutures inside the vagina absorbable catgut is preferable. It is a great annoyance, and may be harmful to have to remove sutures from the cervix or vaginal walls. It is usually unnecessary even to pass a catheter; if the patient can pass urine naturally she should do so, the parts being gently syringed with an antiseptic after each urination.

In amputating the cervix it must be remembered that the supra-vaginal portion is the part affected. Therefore we must separate the bladder in front and the peritoneum behind until the desired level is reached. The section of the cervix must be made in such a manner that the vaginal walls can be easily sutured to the cervical muscle and mucous membrane. After dissecting the bladder off the prolapsed vaginal wall, that viscus must be retained in its place by sutures which bring together the paravaginal connective tissues before cutting away redundant vaginal wall. The excision of an oval piece from the anterior vaginal wall and a purse-string suture after the method of Stolz is an effective way of doing this. The methods of performing posterior colporrhaphy and perineorrhaphy are well known, but they must be thorough and far reaching. It is often necessary to separate the rectum from the vagina almost up to the cervix before the desired result is obtained. The connective tissues beside the rectum may be brought together first so as to retain the rectum in its normal place before suturing the posterior vaginal wall. Carried out in this manner very serious cases of prolapse can be dealt with successfully, but the whole sequence must be looked upon as necessary; the operation must be approached as if it were major surgery, as it is in reality; and no detail must be omitted. Finally, at least two months' rest must be allowed for the cicatrices to consolidate. During this time, if the patient must stand upright a firm perineal pad and binder should be worn.

